# The *Pseudomonas aeruginosa* Orphan Quorum Sensing Signal Receptor QscR Regulates Global Quorum Sensing Gene Expression by Activating a Single Linked Operon

**DOI:** 10.1128/mBio.01274-18

**Published:** 2018-08-28

**Authors:** Fengming Ding, Ken-Ichi Oinuma, Nicole E. Smalley, Amy L. Schaefer, Omar Hamwy, E. Peter Greenberg, Ajai A. Dandekar

**Affiliations:** a Department of Medicine, University of Washington, Seattle, Washington, USA; b Department of Respiratory and Critical Care Medicine, Shanghai General Hospital, Shanghai Jiao Tong University School of Medicine, Shanghai, China; c Department of Microbiology, University of Washington, Seattle, Washington, USA; d Department of Bacteriology, Osaka City University Graduate School of Medicine, Osaka, Japan; Cornell University

**Keywords:** acyl-homoserine lactone, antiactivation, sociomicrobiology

## Abstract

Pseudomonas aeruginosa uses two acyl-homoserine lactone signals and two quorum sensing (QS) transcription factors, LasR and RhlR, to activate dozens of genes. LasR responds to *N*-3-oxo-dodecanoyl-homoserine lactone (3OC12-HSL) and RhlR to *N*-butanoyl-homoserine lactone (C4-HSL). There is a third P. aeruginosa acyl-homoserine-lactone-responsive transcription factor, QscR, which acts to dampen or delay activation of genes by LasR and RhlR by an unknown mechanism. To better understand the role of QscR in P. aeruginosa QS, we performed a chromatin immunoprecipitation analysis, which showed this transcription factor bound the promoter of only a single operon of three genes linked to *qscR*, PA1895 to PA1897. Other genes that appear to be regulated by QscR in transcriptome studies were not direct targets of QscR. Deletion of PA1897 recapitulates the early QS activation phenotype of a QscR-null mutant, and the phenotype of a QscR-null mutant was complemented by PA1895-1897 but not by PA1897 alone. We conclude that QscR acts to modulate quorum sensing through regulation of a single operon, apparently raising the QS threshold of the population and providing a “brake” on QS autoinduction.

## IMPORTANCE

Quorum sensing, a cell-cell communication system, is broadly distributed among bacteria and is commonly used to regulate the production of shared products. An important consequence of quorum sensing is a delay in production of certain products until the population density is high. The bacterium Pseudomonas aeruginosa has a particularly complicated quorum sensing system involving multiple signals and receptors. One of these receptors, QscR, downregulates gene expression, unlike the other receptors in P. aeruginosa. QscR does so by inducing the expression of a single operon whose function provides an element of resistance to a population reaching a quorum. This finding has importance for design of quorum sensing inhibitory strategies and can also inform design of synthetic biological circuits that use quorum sensing receptors to regulate gene expression.

## INTRODUCTION

Pseudomonas aeruginosa is an opportunistic pathogen of humans that uses a process called quorum sensing (QS) to regulate gene transcription in response to cell density (1, 2). The P. aeruginosa genome encodes two complete acyl-homoserine lactone (AHL) QS systems: the LasR-LasI system and the RhlR-RhlI system. LasI and RhlI are signal synthases that produce *N*-3-oxo-dodecanoyl-homoserine lactone (3OC12-HSL) and *N*-butanoyl-homoserine lactone (C4-HSL), respectively (1, 3–6). As cell densities increase, the concentrations of these signals also increase; binding of 3OC12-HSL to LasR and of C4-HSL to RhlR activates both of these transcription factors, and together, LasR and RhlR regulate the transcription of hundreds of genes. LasR and RhlR are homologues of the canonical LuxR QS transcription factor of Vibrio fischeri (7, 8). In the well-studied strain P. aeruginosa PAO1, LasR and RhlR are arranged hierarchically: LasR regulates RhlR transcription, so RhlR activity usually requires both the LasR-LasI system and adequate concentrations of C4-HSL (1).

There is a third AHL-responsive LuxR homologue in P. aeruginosa called QscR (9). Unlike LasR and RhlR, QscR does not have a paired signal synthase; instead, it binds to 3OC12-HSL and to several other long-chain AHLs (10). QscR appears to have a counterregulatory role in P. aeruginosa QS; deletion of QscR results in an acceleration of LasR activation (9, 11), as measured by earlier synthesis of the signals 3OC12-HSL and C4-HSL and the phenazine pyocyanin. Consistent with the early QS activation phenotype, a QscR-null mutant is hypervirulent compared to its parent in a fruit fly model (9).

A transcriptome analysis of a QscR-null mutant compared to the wild type showed 424 genes that were regulated (directly or indirectly) by QscR (12). This regulon substantially overlapped the previously defined QS regulon of P. aeruginosa (13, 14), with the notable difference that QscR appeared to repress many genes that were activated by LasR or RhlR. QscR is one of a few factors that retard expression of LasR- or RhlR-activated genes in P. aeruginosa, described as antiactivators of QS (11). The antiactivator proteins QteE and QslA appear to physically disrupt the ability of LasR and RhlR to induce gene transcription (11, 15). Unlike QscR, however, these proteins are not homologous to LasR or RhlR and do not bind AHL signals.

**FIG 1:**
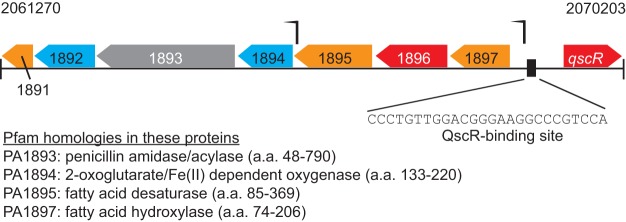
Genomic orientation of *qscR* and PA1891-1897. The *qscR* gene (PA1898) is adjacent to the operon that it regulates, PA1895-1897, although they are carried on opposite strands (43). The genomic coordinates of the *qscR*-binding sequence are at bases 2068990 to 2069015 and lie 262 bp upstream of the coding sequence for PA1897 (10). PA1894 is not in frame with PA1895 and has its own transcriptional start site (19). Arrows indicate the transcription start sites. Red indicates proteins predicted to be in the cytoplasm, orange indicates proteins predicted to be cytoplasmic membrane proteins, and gray indicates periplasmic proteins. The cellular locations of products of the genes shaded in blue are unknown. The schematic was adapted from the pseudomonas.com database (44). Homology to known protein motifs is as reported in the Pfam database (pfam.xfam.org/) (45); there is no identifiable motif in PA1891, PA1892, or PA1896. a.a., amino acids.

The mechanism by which QscR impacts the timing of AHL QS in P. aeruginosa is not known. Several hypotheses have been advanced. A prior study suggested that QscR acts by modulating levels of *lasI* (9). QscR could act by sequestering signal away from LasR. It has been reported that, when overexpressed *in* Escherichia coli, QscR forms inactive heterodimers with LasR (16), which could delay QS gene activation. We hypothesized that QscR, like LasR and RhlR, has a distinct regulon. QscR has been shown to bind to the promoter of the P. aeruginosa 1897 (PA1897) gene, which is immediately adjacent to and transcribed divergently from *qscR* (PA1898) (Fig. 1), and a rubredoxin reductase gene, PA5351 (10). In QS transcriptome studies, PA1897 and PA5351 have been reported to be 3OC12-HSL-regulated genes (12, 13), but they are not regulated by LasR (10).

The P. aeruginosa QS system is complex, with multiple inputs and transcription factors (13). We are interested in the regulation of the P. aeruginosa QS systems and mechanisms that synchronize and stabilize QS in populations. We tested our hypothesis that QscR has a distinct regulon by performing chromatin immunoprecipitation sequencing (ChIP-seq) to identify direct targets of 3OC12-HSL-bound QscR. Surprisingly, we found that QscR bound only to the promoter of PA1897, the first gene of an operon containing the genes PA1895 to PA1897 (PA1895-1897), and we describe experiments showing that the products of these genes, not QscR itself, are the mediators of QS antiactivation.

## RESULTS

### ChIP-seq and direct-binding assays reveal a single target of QscR in the P. aeruginosa genome.

To identify possible binding sites of QscR within the genome of PAO1, we performed a chromatin immunoprecipitation assay using a purified anti-QscR antibody, followed by next-generation sequencing of the DNA bound to QscR. QscR is produced at very low levels in strain PAO1, and we were unable to reliably detect QscR-DNA complexes from the wild type using the ChIP-seq approach. Therefore, we expressed *qscR* under the control of an arabinose-inducible promoter in a *lasR rhlR qscR* triple deletion (3R) mutant and compared DNA sequences captured by an anti-QscR antibody from this mutant (at an optical density at 600 nm [OD_600_] of 1.8) to those captured from a QscR-null mutant containing the vector only.

Our ChIP-seq analysis revealed a single region of the genome to which QscR appeared to bind, in the intergenic region between *qscR* and the adjacent PA1897 gene (*P* = <1 × 10^−10^, *P* score = >145) (Fig. 1). This promoter region has previously been identified as a target of QscR (10) with a QscR-binding site defined at −54 to −29 bp from the PA1897 transcription start site. We did not identify PA5351 in this ChIP-seq experiment, possibly reflecting the previous finding that the level of PA5351 promoter binding was substantially lower than that of PA1897 (10). Our finding of only a single QscR genome target was surprising; however, false-negative results are a consideration in any ChIP-seq analysis.

**FIG 2:**
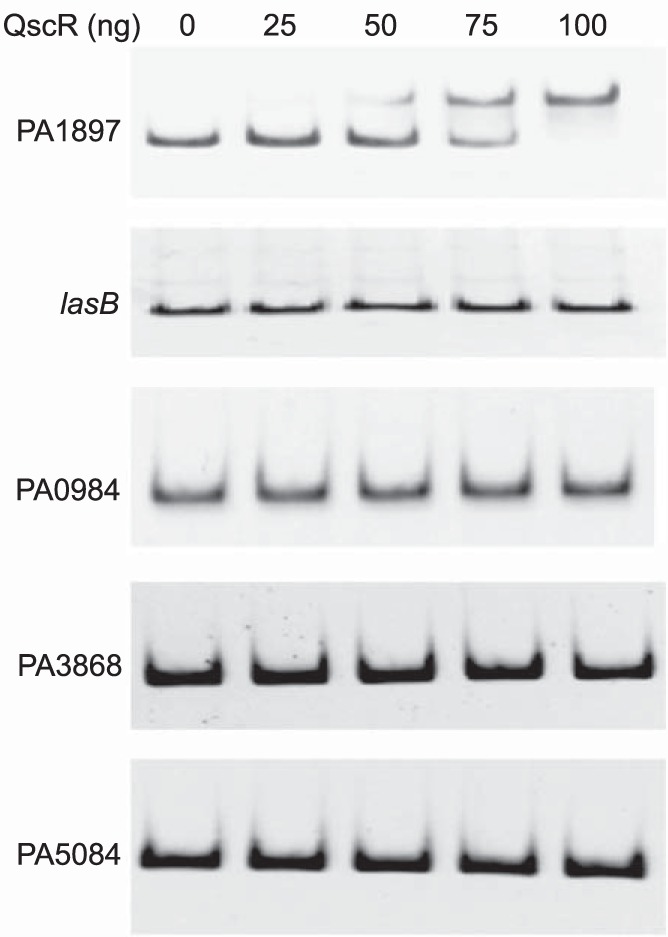
Electrophoretic gel shift assays of potential QscR binding targets. We performed electrophoretic mobility shift assays as described in Materials and Methods using purified QscR and PCR products from promoter regions with visual peaks in our ChIP-seq analysis. A shift occurred only with the PA1897 promoter. The *lasB* promoter, regulated by LasR, is shown as a negative control.

To probe for additional QscR targets not revealed by the ChIP-seq analysis, we performed electrophoretic mobility shift assays (EMSAs) with purified QscR and several promoters that have been shown to be repressed in a QscR transcriptomic analysis (12). We selected from this list those genes that were most likely to have a binding site for QscR, based on previous descriptions of LuxR-homologue binding targets (17). In addition, we asked if QscR could bind to the promoter of the LasR-regulated genes *lasI* (which encodes the 3OC12-HSL signal synthase) and *lasB* (elastase). As expected and predicted by the ChIP-seq results, addition of QscR to the promoter of PA1897 resulted in a DNA gel shift, consistent with binding of QscR to this promoter target, as previously reported (10) (Fig. 2). We did not observe a change in the electrophoretic mobility of DNA fragments containing other promoters (Fig. 2), supporting the idea that QscR does not affect transcription of these genes, including the LasR-regulated *lasI* and *lasB* genes, through direct binding to their promoters.

### Deletion of the PA1895-1897 operon phenocopies a QscR-null mutant.

The ChIP-seq and EMSA results led us to hypothesize that the QscR counterregulation of QS occurs by activation of genes transcribed from the PA1897 promoter. Although transcription of QscR itself has been reported to be activated through this promoter (18), we asked if a PA1897 gene deletion, or a deletion of the operon containing PA1897, might phenocopy a QscR mutant.

The genomic region around PA1897 contains seven genes, PA1891-1897. PA1891-1894 are in a reading frame different from PA1895-1897, and there is a separate transcription start site for PA1894 (determined in P. aeruginosa PA14) (19) (Fig. 1). Whether or not they are in the same operon, PA1894, PA1895, and PA1897 have been reported to be regulated by QS (13). PA1895-1897 code for unknown proteins, although PA1895 and PA1897 have Pfam domains that suggest a role in fatty acid synthesis (Fig. 1).

**FIG 3:**
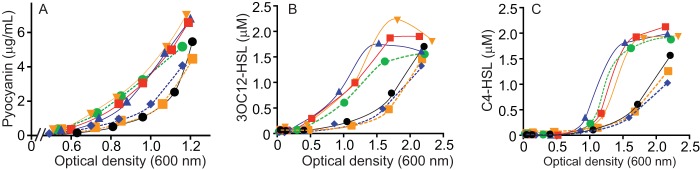
Deletion of PA1897 or of the operon containing PA1897 results in early activation of QS-controlled genes. Deletion of PA1897 or PA1895-1897 phenocopies the *qscR* deletion, but expression of PA1897 alone is insufficient to complement the PA1895-1897 mutant. Time courses of pyocyanin (A), 3OC12-HSL (B), and C4-HSL (C) production are presented. In all panels, wild-type PAO1 is indicated by black circles, the QscR-null mutant by red squares, the PA1897-null mutant by blue triangles, and the PA1895-1897 mutant by inverted orange triangles. The PA1897-null mutant complemented with PA1897 is shown by the dashed blue line and the PA1895-1897 mutant complemented with the full operon by the dotted orange line. The PA1895-1897 mutant complemented with PA1897 alone is shown by the dashed green line.

Initially, we asked if a PA1897-null mutant or a PA1895-1897 deletion mutant (constructed to preserve the PA1894 transcriptional start site and promoter; see Table S1 in the supplemental material) would have the same phenotype of early QS gene activation as a QscR mutant. Deletion of *qscR* accelerates production of 3OC12-HSL and C4-HSL and the phenazine pyocyanin (9), although the final concentrations of all three were similar to those seen with the wild type. We found that both the PA1897 and PA1895-1897 mutants had enhanced pigmentation in mid-logarithmic-phase cultures (Fig. 3A) but the mutations did not affect growth. We observed a similar phenomenon with AHL signal production: both the PA1895-1897 and PA1897 deletion mutants exhibited early 3OC12-HSL and C4-HSL production compared to the wild type (Fig. 3B and C). Expression of a plasmid-borne PA1895-1897 operon with its native promoter was sufficient to complement the operon deletion mutant; however, expression of the PA1897 gene alone could not complement the PA1895-1897 mutation (Fig. 3), strongly supporting the idea that expression of all three genes in the PA1895-1897 operon, and not expression of PA1897 alone, is required for QscR to retard QS gene activation.

10.1128/mBio.01274-18.1 TABLE S1  Bacterial strains and plasmids used in this study. Download TABLE S1, DOCX file, 0.03 MB. Copyright © 2018 Ding et al. 2018 Ding et al. This content is distributed under the terms of the Creative Commons Attribution 4.0 International license.

### A *qscR* mutation can be complemented by a PA1895-1897 expression vector.

**FIG 4:**
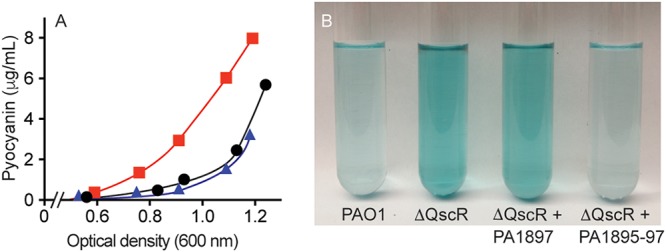
The early QS activation phenotype of a QscR-null mutant is complemented by PA1895-1897 expression vector pJN105-PA1895-97. (A) Time course of pyocyanin production by PAO1 (black circles), a QscR-null mutant complemented with PA1897 alone (red squares), or a QscR-null mutant complemented with PA1895-1897 (blue triangles). (B) Pyocyanin production at an OD_600_ of 1.1. Expression of PA1895-1897 in a QscR mutant is sufficient to reduce pyocyanin production to wild-type levels.

The ChIP-seq data and the data shown in Fig. 3 together suggest that QscR antiactivation of QS is mediated by the PA1895-1897 operon. To test this idea, we transformed a QscR mutant with plasmids containing either the arabinose-inducible PA1895-1897 genes or PA1897 alone. We used an arabinose-inducible promoter because the native QscR-responsive promoter would be ineffective in a QscR mutant background. Consistent with the idea that QscR activation of PA1895-1897 is the primary means by which this transcription factor impedes QS gene activation, arabinose-induced expression of PA1895-1897 was able to fully complement the QscR mutant. Expression of PA1895-97 in a QscR-null mutant reduced pyocyanin production at 12 h to the low, wild-type level (Fig. 4). Finally, and similarly to the experiments described above, pyocyanin production in the *qscR* mutant was not complemented by expression of PA1897 alone (Fig. 4). Together, these findings are consistent with the idea that the antiactivation phenotype of QscR relies upon its regulation of PA1895-1897.

### The PA1897 gene product does not appear to modify the 3OC12-HSL signal directly.

A prior study showed that the QscR transcriptome substantially overlaps the LasR and RhlR regulons (12). Where LasR and RhlR activate many genes, QscR represses many of them (12, 13). Another study suggested that QscR acted to modulate LasI, although the mechanism was unresolved (9). One possible explanation, consistent with these studies and our findings, is that the PA1895-1897 gene products affect 3OC12-HSL levels, either by interfering with synthesis or by modifying the synthesized signal. LasR is highly specific for the 3OC12-HSL signal. Even small modifications to the chemical structure of the signal (including but not limited to reduction of the oxo-group or chain length changes) result in little LasR-dependent activity (10). Little is known about the products of PA1895, PA1896, and PA1897 (Fig. 1). We have not identified homologous proteins in any organism. Results of a Pfam search indicate that PA1897 possibly contains a fatty acid hydroxylase domain and that PA1895 may have a fatty acid desaturase domain. There are no putative functions for PA1896.

**FIG 5:**
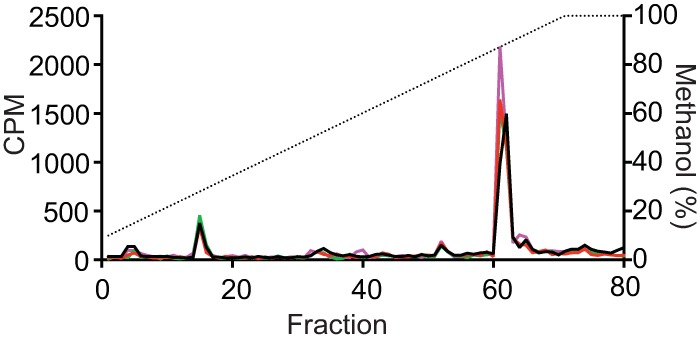
The PA1895-1897 gene products do not appear to chemically modify AHL signals. HPLC profiles of P. aeruginosa ^14^C-AHLs after 2 h of incubation in the presence of PAO1 *ΔPA1895-1897* pJN105.PA1895-97 (red), PAO1 *ΔPA1895-1897* pJN105 vector control (black), or a PPM control (purple). The *x* axis indicates the fraction numbers that were collected over a 10% to 100% methanol-in-water gradient. The left *y* axis denotes the counts per minute (CPM) of radiolabel in each sample fraction (colored solid lines), and the right *y* axis indicates the methanol concentration of the HPLC run (dashed line). Synthetic C4-HSL and 3OC12-HSL compounds eluted in fraction 15 and fractions 61/62, respectively. Radioactivity eluted in the column void volume (fractions 4/5) is presumed to represent unincorporated methionine.

One of the simplest mechanisms by which the PA1895-1897 operon could mediate a delay in QS gene activation would be through AHL signal modification or degradation, comparably to the lactonase AiiA from Bacillus thuringiensis (20) or to the stationary-phase-expressed acylases of P. aeruginosa (21). To test the hypothesis that PA1895-1897 gene products modify 3OC12-HSL such that it no longer functions as a signal for LasR, we incubated P. aeruginosa cells expressing the PA1895-1897 operon with ^14^C-radiolabeled AHL (^14^C-AHL) signals (synthesized as described in Materials and Methods). After 2 h of incubation, the ^14^C-AHLs were subjected to solvent extraction, separated by high-performance liquid chromatography (HPLC), and quantitated by scintillation counting. Even though cells expressing PA1895-1897 were delayed in pyocyanin production relative to the vector controls (confirming that the expressed gene products were functional), there was no difference observed in ^14^C-AHL HPLC elution behavior (Fig. 5). These data suggest that neither 3OC12-HSL nor C4-HSL is degraded by the PA1895-1897 gene products, although we cannot exclude the possibility that they cause subtle modifications in the AHL compounds that do not alter the HPLC elution behavior.

## DISCUSSION

QS in Pseudomonas aeruginosa is complex and involves several transcription factors that variously coregulate, repress, or activate genes (1, 8, 22, 23). This bacterium has evolved several counterregulatory mechanisms to control QS activation both early and late in growth, possibly because there are so many interacting QS circuits in P. aeruginosa. As mentioned above, the proteins QteE and QslA interact physically with LasR and RhlR to impair early activation of QS-regulated genes (11, 15). In the stationary phase, acylases (21) and the regulatory element *rsaL* (24) provide a check on uncontrolled QS gene activation.

Our experiments with QscR demonstrate a third means by which quorum sensing can be restrained, one that both depends on production of the QS signal 3OC12-HSL and impairs the LasR-signal interaction by an as-yet-unresolved mechanism. We showed that QscR regulation of a single P. aeruginosa operon, PA1895-1897, accounts for the antiactivation phenotype. Deletion of this operon results in phenotypes that are the same as those exhibited by a QscR deletion mutant. This operon encodes three proteins that somehow act to delay the timing of QS induction.

Therefore, at low cell densities, production of 3OC12-HSL has the paradoxical effect of inducing a set of gene products involved in disrupting LasR QS. As a consequence, QscR would effectively increase the activation threshold for LasR QS in P. aeruginosa. However, because QscR is not substantially induced by either its own interactions with signal or by LasR (13), once positive autoactivation of *lasI* has occurred, it is unlikely that QscR-PA1895-97 modulation of 3OC12-HSL levels would have a material impact on QS.

We do not know how expression of the gene products encoded by PA1895-1897 alters signal production in P. aeruginosa. Although we did not find evidence that the gene products modify signal *per se*, this kind of enzymatic activity is still possible, especially if it occurs inefficiently or the modification is insufficient to alter HPLC elution behavior of the signal. Alternatively, the products of PA1895-1897 (or PA1891-1897) might together synthesize an antagonist to LasR. Other possibilities are that the products of PA1895-1897 interact with LasI, the 3OC12-HSL signal synthase, or that they catalyze reactions that reduce AHL substrate pools, so that cells simply produce less signal.

The presence of this kind of resistance element within the P. aeruginosa QS circuitry might create a bistable system where, for a given range of signal concentrations, QS may be “on” or “off” depending on the prior state of the population (25). The presence of bistability in a system requires resistance to activation, a positive-feedback loop, and prevention of overactivation of the system (25). The ability of the QscR circuit to antiactivate QS by reduction in signal concentrations directly provides resistance to activation of LasR and, therefore, AHL QS in P. aeruginosa. The other necessary elements of a bistable system in P. aeruginosa are provided by autoinduction of *lasI* and by stationary-phase dampers of QS, including *rsaL* (11, 24). Additionally, resistance to activation provided by QscR might facilitate synchronization of QS activation (26), as the concentration of signal required for activation would be higher than in a QscR mutant.

QscR recognizes and is activated by several AHL signals, including C10-HSL, 3OC10-HSL, C12-HSL, and 3OC12-HSL (10). Binding of QscR to any of these signals likely results in expression of the PA1895-1897 genes. Although our experiments did not test whether QscR-regulated expression of PA1895-1897 might have an impact on P. aeruginosa interactions with other species, it seems likely that it can do so, particularly if the other species produces QscR-binding signals (18). QS activation has been implicated in intraspecies competition (27–30), and P. aeruginosa may gain a competitive advantage over other species by slightly delaying QS activation, if competitive factors are quorum regulated (particularly if a certain cell density is required for the effectiveness of the competitive factors). This possibility that QscR could detect signals produced by other bacteria might explain why it, uniquely among the P. aeruginosa LuxR homologues, has broad signal specificity (10).

Our results demonstrate an added layer of regulation in P. aeruginosa quorum sensing. P. aeruginosa uses QS to directly or indirectly regulate the expression of several hundred genes (13). Because of this metabolic cost of QS, it is likely to result in a fitness disadvantage for cells that activate the circuit earlier than the rest of the population. QscR directly regulates a single operon that codes for proteins which somehow interfere with QS activation. In addition to previously described mechanisms of antiactivation (11), our finding reinforces the idea that prevention of early QS activation is critically important and emphasizes the exquisite level of regulatory control required for QS in P. aeruginosa.

## MATERIALS AND METHODS

### Bacterial strains, plasmids, and culture conditions.

The bacterial strains and plasmids used in this study are listed in Table S1 in the supplemental material. For all experiments, either P. aeruginosa or E. coli was grown at 37°C with shaking (250 rpm). Unless otherwise indicated, Luria-Bertani (LB) broth buffered with 50 mM 3-(N-morpholino)propanesulfonic acid (MOPS) was used. Media were supplemented with antibiotics for selection as follows: for E. coli, ampicillin at 100 µg ml^−1^ and gentamicin at 10 µg ml^−1^; for P. aeruginosa, carbenicillin at 300 µg ml^−1^ and gentamicin at 100 µg ml^−1^. Deletion mutants of P. aeruginosa were constructed by homologous recombination as previously described (31). To generate plasmids for homologous recombination, we used the method of Kostylev et al. (32). This technique requires the production of overlapping PCR products, including that of a plasmid, which are assembled *in vivo* by E. coli DH5α. Primers used for the generation of these constructs are listed in Table S2.

10.1128/mBio.01274-18.2 TABLE S2  Primers used in this study. Download TABLE S2, DOCX file, 0.02 MB. Copyright © 2018 Ding et al. 2018 Ding et al. This content is distributed under the terms of the Creative Commons Attribution 4.0 International license.

### ChIP-seq.

Bacterial strains used in this experiment include a P. aeruginosa PAO1 *qscR* deletion mutant and the P. aeruginosa *lasR rhlR qscR* triple deletion (3R) mutant. The *qscR* overexpression plasmid pJN105-QscR (10) was introduced into the 3R mutant. PAO1 Δ*qscR* and 3R-pJN105-QscR were incubated with 2 µM 3OC12-HSL in LB-MOPS (pH 7.0) at 37°C with shaking. For the 3R strain containing pJN105-QscR, 20 µg/ml gentamicin, with or without 0.01% arabinose, was added for plasmid maintenance. Growth was monitored as optical density at 600 nm.

A chromatin immunoprecipitation assay was performed for P. aeruginosa as previously described for LasR (33). Bacteria were grown in 40 ml of LB-MOPS to an OD_600_ of 1.8. DNA was cross-linked to transcription factors with 1% formaldehyde and fragmented using a Branson microtip sonicator (three times for 10 s each time at an output of 0.4). The size of the fragments was confirmed to be in the 300-bp to 1,000-bp range by agarose gel electrophoresis. Fragmented DNA was immunoprecipitated using a 50% protein A Sepharose slurry (nProtein A 4 Fast Flow; Amersham Biosciences) with purified anti-QscR antibody (see below). Cross-links between the protein and DNA were reversed by incubating the eluate at 65°C overnight. DNA was purified using phenol-chloroform-isoamyl alcohol extraction and ethanol precipitation and was then further purified using a Monarch PCR and DNA cleanup kit (New England Biolabs). DNA was sequenced using an Illumina MiSeq system.

### ChIP-seq analysis.

ChIP-seq libraries were prepared according to Illumina protocol and sequenced in a single MiSeq flow cell. ChIP-seq analysis of FASTQ output was performed using the Strand NGS (Bangalore, India) v 3.1.1 ChIP-seq pipeline. Single-end 150-bp reads were aligned to the PAO1 reference genome (RefSeq accession no. NC_002516). Peaks were identified using MACS (model-based analysis of ChIP-seq) peak detection (34) set to default parameters with the PAO1 *qscR* deletion mutant alignment as the control.

### Preparation of purified QscR and affinity-purified anti-QscR antibody.

Purified recombinant QscR was prepared as previously described (35). A polyclonal antiserum against QscR was obtained from rabbits immunized with purified recombinant QscR. An affinity column, in which up to 9 mg of QscR was immobilized, was prepared using an AminoLink Plus immobilization kit (Thermo Fisher Scientific Company). Antibody was preadsorbed with a PAO1 *qscR* deletion mutant (9) grown in 50 ml of LB-MOPS at 37°C overnight. Cells were harvested by centrifugation, suspended in 2 ml of Tris-buffered saline (pH 7.2), and lysed by sonication. After insoluble material was removed by ultracentrifugation at 150,000 × *g* for 30 min, the cleared cell extract was mixed with an equal volume of antiserum and incubated for 4 h on ice. Insoluble aggregates were removed by ultracentrifugation, and the resulting supernatant was subjected to affinity purification with the prepared column. Binding, washing, and elution were performed according to the manufacturer’s instructions. The quality and specificity of the purified antibody were assessed by Western blotting.

### Electrophoretic mobility shift assays.

We used Regulatory Sequence Analysis Tools (RSAT; http://embnet.ccg.unam.mx/rsat/) to search for promoter sequences with a conserved CT-N12-AG binding motif for LuxR-type transcription factors (36). Primers (Table S2) were designed to amplify 150-bp to 300-bp DNA fragments as probes to assess QscR binding in EMSAs. These probes were generated by PCR amplification with PAO1 genomic DNA as a template. The PCR-amplified probes (40 ng) were mixed with purified QscR protein (0 to 100 ng) in a 10-µl reaction mixture containing 20 mM Tris (pH 7.5), 50 mM KCl, 10% glycerol, 1 mM dithiothreitol, and 20 µM 3OC12-HSL. After 20 min at room temperature, the samples were separated by electrophoresis on a 5% nondenaturing acrylamide-Tris-glycine-EDTA gel in Tris-glycine-EDTA buffer at 4°C. Gels were soaked in 10,000-fold-diluted SYBR green I nucleic acid stain (catalog no. S7585; Invitrogen, Eugene, OR, USA), and DNA was visualized under UV light at 300 nm.

### Pyocyanin measurements.

Pyocyanin assays were performed with P. aeruginosa grown in a pyocyanin production medium (PPM), which was composed of a pancreatic digest of gelatin (20.0 g), magnesium chloride (1.4 g), and potassium sulfate (10.0 g) in 1 liter of purified water containing 10 g of glycerol per liter (37). Pyocyanin was extracted from 5 ml culture fluid with 2 ml chloroform and was then extracted from the chloroform with 1 ml 0.2 M hydrochloric acid–water. The absorbance was measured at 520 nm, and this value was converted to milligrams per milliliter of pyocyanin by multiplying the optical density at 520 nm (OD_520_) by 17.072 (38).

### AHL measurements.

Concentrations of 3OC12-HSL were measured using a biological assay as described previously (10). To measure C4-HSL concentrations, we developed an mCherry reporter strain. This strain contains *rhlR* under the control of the *tac* promoter and a P*rhlA*-mCherry fusion. The reporter strain was grown overnight and was subcultured to an OD_600_ of 0.025 into minimal A medium (5) with extracted AHLs. After 18 h, the samples were loaded into a 96-well plate (Costar assay; Corning Incorporated, Kennebunk, ME) and fluorescence was measured in a BioTek Synergy H1 plate reader (excitation, 587 nm; emission, 620 nm). Synthetic 3OC12-HSL and C4-HSL (Cayman Chemical) were used to generate standard curves.

### Radiotracer assay.

To obtain ^14^C-labeled 3OC12-HSL and C4-HSL compounds, we used a radiolabeling protocol that was described previously (39, 40). P. aeruginosa PAO1 cells were grown with shaking at 37°C in Jensen’s medium–0.3% glycerol (41) to mid-log phase. A 10-ml aliquot of cells was incubated (90 min) with 10 µCi of l-[1-^14^C]methionine (American Radiolabeled Chemicals, St. Louis, MO), a portion of which is converted to *S*-adenosylmethionine, a substrate for AHL synthesis. To harvest ^14^C-AHLs, cells were removed by centrifugation and the cell-free supernatant was extracted twice with equal volumes of acidified ethyl acetate (EtAc). To test if the PA1895-1897 gene products modified ^14^C-AHLs, the *ΔPA1895-1897* mutant harboring arabinose-inducible plasmid pJN105.PA1895-97 was inoculated into fresh PPM plus gentamicin and 0.05% arabinose and grown for 6 h (to an OD_600_ of between 0.7 and 0.8) at 37°C with shaking. A 5-ml culture aliquot was transferred to a 15-ml polypropylene tube containing 1/20 of the total ^14^C-AHL extract (prepared as described above; solvent removed under a gentle stream of N_2_ gas) and incubated at 37°C with shaking for 2 h. ^14^C-AHLs were reextracted twice with equal volumes of EtAc and evaporated to dryness. Approximately 30% of this extract was separated by C_18_ reverse-phase HPLC in a 10% to 100% methanol gradient with a flow rate of 0.75 ml/min as described previously (42). Fractions were collected at 1-min intervals and mixed with 4 ml of counting cocktail (3a70b; Research Products International), and radioactivity was determined by liquid scintillation counting. For comparison, the same protocol was performed using the *ΔPA1895-1897* mutant with pJN105 vector control and PPM broth.

### Accession number(s).

Sequencing data from this study were deposited in the NCBI Sequence Read Archive under accession number SRP149739 and in BioProject under accession number PRJNA472067.
