# Supplementary material for: The Pseudomonas aeruginosa Orphan Quorum Sensing Signal Receptor QscR Regulates Global Quorum Sensing Gene Expression by Activating a Single Linked Operon
Source: mBio. 2018 Aug 28;9(4):e01274-18. doi: 10.1128/mBio.01274-18 (PMC6113619; doi:10.1128/mBio.01274-18)
Supplement: TABLE S1 [file mbo004184035st1.docx]

Supplemental table 1. Bacterial strains and plasmids used in this study

Bacterial strain or plasmid Description Reference or source

*P. aeruginosa*PAO1 Wildtype (1)

PAO1 Δ*qscR* PAO1 containing an unmarked, in-frame *qscR* deletion This work

PAO1 Δ*3R* PAO1 containing unmarked, in-frame deletions of *lasR*, *rhlR*, and *qscR* (2)

PAO1 Δ*PA1897* PAO1 containing an unmarked, in-frame PA1897 deletion This work

PAO1 Δ*PA1895-1897* PAO1 containing an unmarked, in-frame deletion of PA1895-PA1897

from genome coordinates 2065802 to 2068725 This work

Other strains

*E. coli* DH5α F^-^ Φ80*lac*ZΔM15 Δ(*lac*ZYA-*arg*F) U169 *rec*A1 *end*A1 *hsd*R17(r_k_^-^, m_k_^+^)  Invitrogen
 *pho*A*sup*E44 *thi*-1 *gyr*A96 *rel*A1 λ^-^

Plasmids

pJN105 *araC*-P_BAD_ cassette cloned in pBBR1MCS-5, gentamicin resistance (Gm^r^) (3)

pJN105.qscR Arabinose-inducible *qscR* in pJN105, Gm^r^  (4)

pJN105.PA1897 Arabinose-inducible PA1897 in pJN105, Gm^r^ This work

pJN105.PA1895-97 Arabinose-inducible PA1895-1897 in pJN105, Gm^r^ This work

pBBR1MCS-5 Moderate copy number plasmid, Gm^r^  (5)

pBBR.PA1897 pBBR1MCS5 containing PA1897 (native promoter), Gm^r^ This work

**REFERENCES**

1. Stover CK, Pham XQ, Erwin AL, Mizoguchi SD, Warrener P, Hickey MJ, Brinkman FS, Hufnagle WO, Kowalik DJ, Lagrou M, Garber RL, Goltry L, Tolentino E, Westbrock-Wadman S, Yuan Y, Brody LL, Coulter SN, Folger KR, Kas A, Larbig K, Lim R, Smith K, Spencer D, Wong GK, Wu Z, Paulsen IT, Reizer J, Saier MH, Hancock RE, Lory S, Olson MV. 2000. Complete genome sequence of *Pseudomonas aeruginosa* PAO1, an opportunistic pathogen. Nature 406:959-64.

2. Chugani S, Greenberg EP. 2010. LuxR homolog-independent gene regulation by acyl-homoserine lactones in *Pseudomonas aeruginosa*. Proc Natl Acad Sci USA 107:10673-8.

3. Newman JR, Fuqua C. 1999. Broad-host-range expression vectors that carry the L-arabinose-inducible *Escherichia coli* araBAD promoter and the araC regulator. Gene 227:197-203.

4. Lee JH, Lequette Y, Greenberg EP. 2006. Activity of purified QscR, a *Pseudomonas aeruginosa* orphan quorum-sensing transcription factor. Mol Microbiol 59:602-609.

5. Kovach ME, Elzer PH, Hill DS, Robertson GT, Farris MA, Roop RM, 2nd, Peterson KM. 1995. Four new derivatives of the broad-host-range cloning vector pBBR1MCS, carrying different antibiotic-resistance cassettes. Gene 166:175-6.
