# Supplementary material for: The Pseudomonas aeruginosa Orphan Quorum Sensing Signal Receptor QscR Regulates Global Quorum Sensing Gene Expression by Activating a Single Linked Operon
Source: mBio. 2018 Aug 28;9(4):e01274-18. doi: 10.1128/mBio.01274-18 (PMC6113619; doi:10.1128/mBio.01274-18)
Supplement: TABLE S2 [file mbo004184035st2.docx]

Supplemental table 2. Primers used in this study

*Primers used in EMSA assays*

PA0984 forward CAG CGT GAG AGT GGT CAA C

PA0984 reverse CAA CGA TTC CCA CCT AGC

pyoS5 forward CCA ATC CCA CTC CTG AAT ATC G

pyoS5 reverse GGA ACC TCG TAT GCG TAT TGA TC

PA0988 forward CAG GTC ACG AAT CCT ACA CG

PA0988 reverse CCA GAA CGA ACG GGA AAG C

PA1897 forward CCA GAA CGA ACG GGA AAG C

PA1897 reverse GTC AGG CGT GGA TAG CTT G

PA2984 forward GTTCTGGCTGTCGTTCCTG

PA2984 reverse CACTACCAGCAGTTCGATCC

PA4908 forward CGACTATCGCCACATCGACATC

PA4908 reverse GAGGTGGTAACGCAACTGACG

PA3865 forward GTG GCT GTG TAT GGC TCA AG

PA3865 reverse GCA GGT TCT TGG TGT TCA G

PA5084 forward GAT CGC CTG GAA CAC TTC G

PA5084 reverse CAC CAG GGT CAC GTC GAA TC

lasB forward GCT TCA CCGAGCAGAACTCC

lasB reverse GGC TGG GCT GGT CCA TGT AG

lasI forward CTC TCG TGT GAA GCC ATT GCT C

lasI reverse GAA CAC TTG AGC ACG CAA C

rhlA forward ACG GTG ATG CTG GTC AAC

rhlA reverse GTG ATT GAC CTC GAA GCG

*Primers used for construction of knockout plamids*

PA1897 upstream forward TCG ACT CTA GAG GAT CCC CGG GGA GGC TGA AGA GAA CTT CTC GAT GAG C

PA1897 upstream reverse CGA TGT CTG GAA TCA TGG CGT TCA CCT AAT CAT ATT TAT CTC CTC TTC CTT AGG

PA1897 downstream forward CCT AAG GAA GAG GAG ATA AAT ATG ATT AGG TGA ACG CCA TGA TTC CAG ACA TCG

PA1897 downstream reverse AAT TAA TTA AGG TAC CGA ATT CGA GCT CGA GAC TCG CTC GAA GGT GTA GC

pEXG2 for PA1897 forward AGC CGC GCT ACA CCT TCG AGC GAG TCT CGA GCT CGA ATT CGG TAC C

pEXG2 for PA1897 reverse GTG CTC ATC GAG AAG TTC TCT TCA GCC TCC CCG GGG ATC CTC TAG AGT CGA C

PA1895-97 upstream forward TAA GCT AAT TCC ACA CAT TAT ACG AGC CGG GGC GCG GTC AGC GGG AAA GG

PA1895-97 upstream reverse CTG GGC AGG TCC TGC ATG AAG GAG AAC ACC CGC ATA TTT ATC TCC TCT TCC TTA GGG

PA1895-97 downstream forward CGT CCG CCC CTA AGG AAG AGG AGA TAA ATA TGC GGG TGT TCT CCT TCA TGC AG

PA1895-97 downstream reverse GGG GAT CCT CTA GAG TCG ACC TGC AGA AGC TTG CCC AGC TCC AGA TAG GCG T

pEXG2 for PA1895-97 forward AGC GAC GGG ATC AAC GCC TAT CTG GAG CTG GGC AAG CTT CTG CAG GTC GA

pEXG2 for PA1895-97 reverse CGG TGC GCG GGC GCC TTT CCC GCT GAC CGC GCC CCG GCT CGT ATA ATG TGT GGA AT
